# Nucleocapsid single point-mutation associated with drop-out on RT-PCR assay for SARS-CoV-2 detection

**DOI:** 10.1186/s12879-023-08707-w

**Published:** 2023-10-23

**Authors:** Fernanda de Mello Malta, Deyvid Amgarten, Alexandre Rodrigues Marra, Roberta Cardoso Petroni, Luiz Henrique da Silva Nali, Ricardo Andreotti Siqueira, Miguel Cendoroglo Neto, Silvia Cassiano Oler, João Renato Rebello Pinho

**Affiliations:** 1 Laboratório Clínico - Hospital Israelita Albert Einstein, Av. Albert Einstein, 627/701, Sao Paulo, SP 05651-901 Brazil; 2 Post-Graduation Program in Health Sciences, Santo Amaro University, Rua Prof. Enéas de Siqueira Neto, 340 - Jardim das Imbuias, Sao Paulo, SP Brazil

**Keywords:** SARS-CoV-2, Viral agent, Genomic data, Molecular tests, Vaccines

## Abstract

**Background:**

Since its beginning, the severe acute respiratory syndrome coronavirus 2 (SARS-CoV-2) pandemic has been a challenge for clinical and molecular diagnostics, because it has been caused by a novel viral agent. Whole-genome sequencing assisted in the characterization and classification of SARS-CoV-2, and it is an essential tool to genomic surveillance aiming to identify potentials hot spots that could impact on vaccine immune response and on virus diagnosis. We describe two cases of failure at the N2 target of the RT-PCR test Xpert® Xpress SARS-CoV-2.

**Methods:**

Total nucleic acid from the Nasopharyngeal (NP) and oropharyngeal (OP) swab samples and cell supernatant isolates were obtained. RNA samples were submitted to random amplification. Raw sequencing data were subjected to sequence quality controls, removal of human contaminants by aligning against the HG19 reference genome, taxonomic identification of other pathogens and genome recovery through assembly and manual curation. RT-PCR test Xpert® Xpress SARS-CoV-2 was used for molecular diagnosis of SARS-CoV-2 infection, samples were tested in duplicates.

**Results:**

We identified 27 samples positive for SARS-CoV-2 with a nucleocapsid (N) gene drop out on Cepheid Xpert® Xpress SARS-CoV-2 assay. Sequencing of 2 of 27 samples revealed a single common mutation in the N gene C29197T, potentially involved in the failed detection of N target.

**Conclusions:**

This study highlights the importance of genomic data to update molecular tests and vaccines.

## Background

SARS-CoV2, the etiological agent of Coronavirus associated Disease 2019 (COVID-19), has infected more than 756.291.327 individuals and has led more than 6.841.640 to death. Although vaccination has reduced considerably the number of cases, nearly half a million cases are still reported daily worldwide [1]. SARS-CoV-2 infection can cause some wide different symptoms or even be an asymptomatic infection for some people. The more common symptoms are fever, cough, and shortness of breath [2].

SARS-CoV-2 is an enveloped virus whose structure contains a single-stranded RNA (ssRNA) genome with around 30 kb, presents a 5’-untranslated region (UTR), a conserved replicase domain (ORF1ab) cleaved into 16 non-structural proteins involved in virus transcription and genome replication, four structural proteins (S, E, M, and N), and accessory proteins (ORF3a, ORF6, ORF7a, ORF7b, ORF8, and ORF10), and a highly conserved 3’-UTR1 [3–5].

Several mutations in SARS-CoV-2 RNA have been described worldwide during this pandemic scenario, which is mostly expected since for more than a year most of the preventive measures were based on non-pharmacological approaches, without any vaccination programs available previously, the high r0 [6] and many susceptible individuals, the virus replicated considerably and several variants were positively selected and later described [7], which have brought further concern for the public health authorities. The mutation emergence dynamics are necessary for the pandemic vigilance, virulence and also for diagnosis, especially the assays based on nucleic acid detection [8]. Importantly, the spike protein mutation D614G became dominant in SARS-CoV-2 during the COVID-19 pandemic. However, the mutational impact on the viral spread and vaccine efficacy remains to be elucidated. D614G variant grows to a higher titer as pseudo-typed virions. In infected individuals, D614G is associated with lower cycle thresholds (Ct) values in real time reverse transcription-polymerase chain reaction (RT-PCR), suggestive of higher upper respiratory tract viral loads, but not with increased disease severity. These findings illuminate important changes for a mechanistic understanding of the virus and support continuing surveillance of Spike mutations to aid with the development of immunological interventions [9]. The prompt detection of new mutations and/or variants is necessary to identify vulnerable points for vaccines update, development of treatments, diagnosis, and more importantly to show the evolution of the virus. Several methods of sequencing the complete genome of SARS-CoV2 have aided the scientific community to identify the mutations that the virus has undergone since the beginning of the pandemic.

Hernandez et al. sequenced and analyzed 457 SARS-CoV-2 collected in Mexico, Guatemala and Belize and identified one hundred and eighty-seven distinct variants, distributed in six genes. Most mutations were found in the ORF1ab gene, followed by the S gene. The N gene was the third gene with the most mutations, although studies showed that the N gene is highly conserved in the coronavirus [10]. The genomic sequences of SARS-CoV-2 in these countries showed similar mutations related to a high infectivity virulent strain related to an increased spread of the virus in the countries evaluated [10]. N Gene is one of the four structural proteins (S, N, E and M) present in the SARS-Cov-2, an essential part for the virus to entry into host cells. Here we describe two cases of failure at the N2 target of the RT-PCR test Xpert® Xpress SARS-CoV-2 (Cepheid®). We hypothesize that the mutation found C29197T is responsible for this failure.

## Methods

### Next-generation sequencing of the viral full-length genome

We extracted total nucleic acid from the Nasopharyngeal (NP) and oropharyngeal (OP) swab samples and cell supernatant isolates with the QIAamp Viral RNA Mini kit (QIAGEN, Hilden, Germany). The purification and concentration steps were carried out with RNA Clean & Concentrator kit (Zymo Research, Irvine, USA) with DNAse I treatment during the concentration process, to improve the viral reads detections and deplete contaminant genomic DNA. Depletion of human ribosomal RNA was performed with the concentrated RNA product using the QIAseq Fast Select RNA Removal kit (QIAGEN). Finally, RNA samples were submitted to random amplification following the methodology described in Greninger et al. (2015) with a few modifications [11]. The preparation of sequencing libraries for the Illumina platform was carried out with the Nextera XT Kit (Illumina, San Diego, USA) using the random two-step PCR amplification product as input, following the kit’s standard instructions. The libraries were quantified after fluorescence measuring with the Qubit instrument (Thermo Fisher Scientific, Waltham, USA) and loaded on the NextSeq 550 equipment (Illumina) for sequencing with MID 300 paired-end reads (Illumina).

### Sequencing analysis

Raw sequencing data were analyzed by a pipeline developed at Hospital Israelita Albert Einstein. In summary, raw sequencing data were subjected to sequence quality controls, removal of human contaminants by aligning against the HG19 reference genome, taxonomic identification of other pathogens and genome recovery through assembly and manual curation. Quality control was performed using Cutadapt software to filter sequences by length (< 50 bp), average quality [12] (Qp < 20) and trim options to remove low-quality ends (9 bp to 5’ end and 5 bp to 3’ end). Reads that passed quality control (QC) were mapped to HG19 human reference genome using Burrow-Wheeler Aligner (BWA) [13] with default parameters. Not mapped reads were submitted to assembly using SPAdes algorithm v1.13 [14]. Contigs were inspected and manually curated using Geneious software to generate a final assembly. The complete genome was compared to SARS-CoV-2 reference and close isolates by multiple sequence alignment. SARS-Cov-2 variants were called using GATK Haplotype caller with zygosity set to 1 and further parameters set to default. The viral reference genome was NC_045512.2, which is the first isolate from Wuhan, China. Annovar [15] was used to annotate variants using an appropriate SARS-CoV-2 database. The curation of variants was carried out by manual inspection of NGS mapping using IGV.

### Patients and RT-PCR detection

The following study was submitted to ethical committee of Hospital Israelita Albert Einstein, Sao Paulo, Brazil. RT-PCR test Xpert® Xpress SARS-CoV-2 (Cepheid®) was used for molecular diagnosis of SARS-CoV-2 infection, samples were tested in duplicates. The Xpert Xpress SARS-CoV-2 test is a real-time RT-PCR test intended for the qualitative detection of nucleic acid from the SARS-CoV-2 in nasopharyngeal swab, nasal swab, or nasal wash/aspirate specimen. Positive results are indicative of the presence of SARS-CoV-2 RNA based on the detection of signal from N2 nucleic acid target or signals for both nucleic acid targets (genes N2 and E).

Each Xpert Xpress SARS-CoV-2 cartridge includes a Sample Processing Control (SPC) and Probe Check Control (PCC). The SPC should be positive in a negative sample and can be negative or positive in a positive sample. Before the start of the PCR reaction, the equipment measures the fluorescence signal from the probes to monitor bead rehydration, reaction tube filling, probe integrity, and dye stability. The PCC passes if it meets the validated acceptance criteria.

## Results

Between October 2020 and January 2021, our laboratory at a major hospital in São Paulo, Brazil, conducted screenings for SARS-CoV-2. During this period, we encountered 27 cases in which only the N2 gene could not be detected by. the Xpert® Xpress SARS-CoV-2 test (Cepheid®). We were able to clinically evaluate and follow the outcomes of two of these cases.

**Fig. 1:**
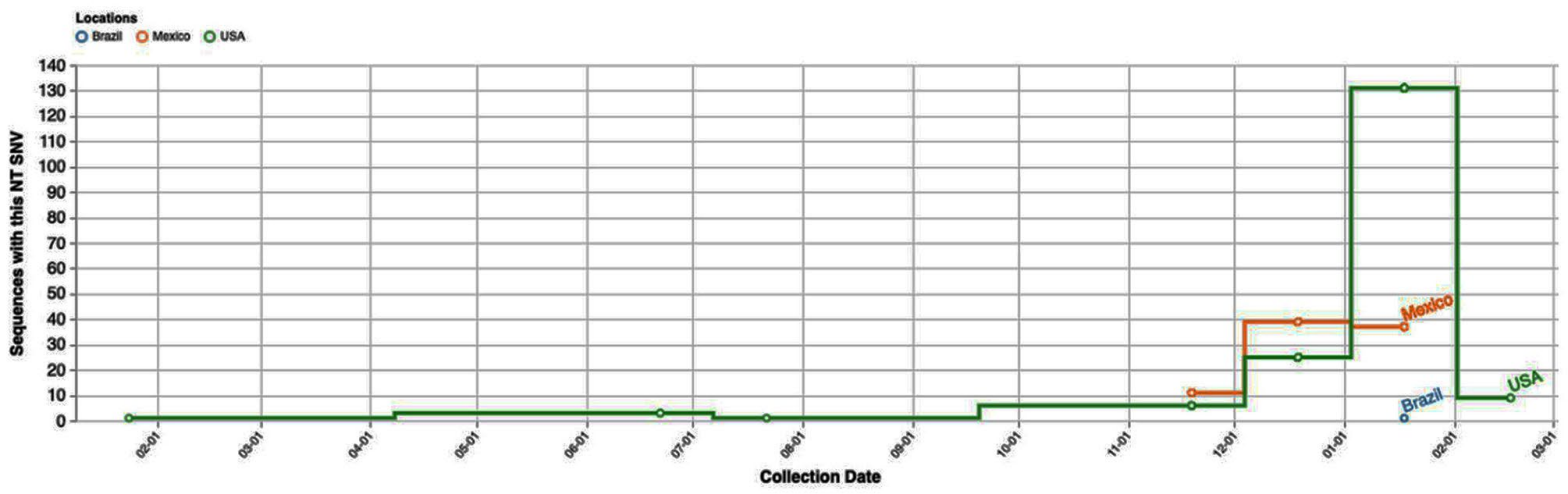
The figure illustrates the number of sequences harboring the described single nucleotide variant over the study period, with a predominant presence observed in the USA and Mexico, as reported in data sourced from the COVID-19 CG website (https://covidcg.org/)

**Fig. 2:**
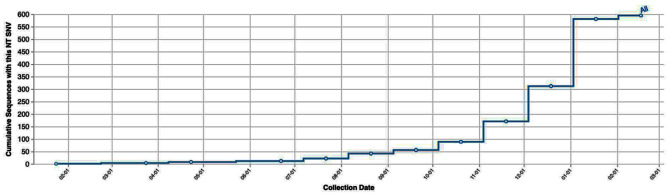
Cumulative number of this SNV cases worldwide during the study period, as reported in data sourced from the COVID-19 CG website (https://covidcg.org/)

Notably, one of these patients had recently traveled to Mexico, where the C29197T Single Nucleotide Variant (SNV) was prevalent at the time of the study. Cases of this particular SNV were also on the rise, as depicted in Figs. 1 and 2.

Case 1 involved a 30-year-old female with no comorbidities who had traveled to Mexico for 11 days. She developed symptoms four days after returning, including periorbital pain, photophobia, nausea, loss of appetite, and hyposmia. Nasopharyngeal and oropharyngeal swabs were collected and tested positive for SARS-CoV-2 using the Xpert® Xpress SARS-CoV-2 assay (Cepheid®). On the fifth day, the patient experienced a single episode of soft stools. She was monitored via telemedicine services at home.

Case 2 was a 52-year-old male with comorbidities like arterial hypertension and overweight, who developed symptoms without any travel history to Mexico or known exposure to infected individuals. Two days later, an RT-PCR Xpert® Xpress SARS-CoV-2 assay was performed, and he was subsequently hospitalized three days later due to dyspnea, fever, and cough. He remained in the ward for eight days with non-invasive oxygen support and was discharged without complications.

These two cases highlight the local circulation of the virus with the identified SNV, as one patient acquired the infection in Brazil without any travel or contact history related to Mexico, where the SNV was prevalent.

**Fig. 3:**
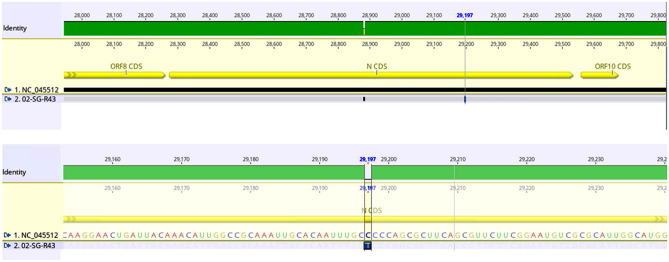
Alignment of the sample sequence against the reference SARS-CoV-2 genome (NC_045512) using Genious software (version 2022). The figure illustrates the presence of the C29197T single nucleotide variant (SNV) within the N gene

We conducted viral genome sequencing and alignment with the reference genome NC_045512. The bioinformatics analysis indicates high-quality sequence metrics, including a Q30 value of 88.9% and 88.4%, mapped reads of 1.6k and 27k, horizontal coverage of 98.3% and 100%, and average coverage of 13x and 228x for Case 1 and Case 2, respectively. The sequencing results confirmed the presence of the mentioned SNV in the SARS-CoV-2 samples that had initially gone undetected by the Xpert® Xpress SARS-CoV2, as shown in Fig. 3.

**Fig. 4:**
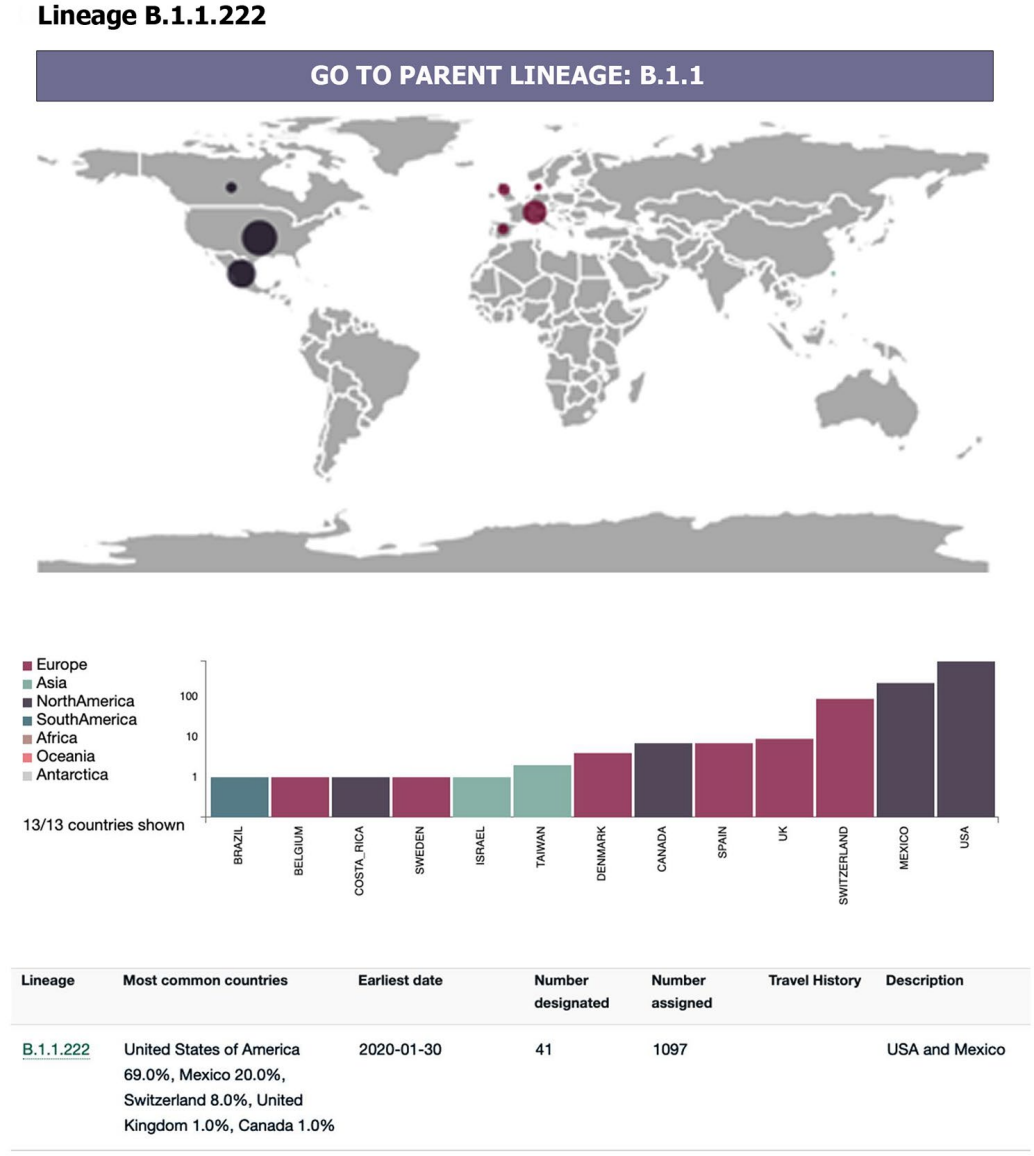
The worldwide distribution of the B.1.222 lineage cases reveals a predominant occurrence in European and North American countries, as indicated by data sourced from the *Pango* lineages website (https://cov-lineages.org/)

Furthermore, phylogenetic analysis indicated that this mutation was part of Lineage B1.1.222 of SARS-CoV-2, which was more commonly found in European and North American countries, as illustrated in Fig. 4.

## Discussion

In this report, we present two cases that were sequenced in our laboratory but failed to detect the N2 gene using the RT-PCR method with the Xpert® Xpress SARS-CoV-2 test. Out of 27 samples, these two cases exhibited a profile of N2 gene detection failure during the sequencing process. It is noteworthy that both patients experienced favorable clinical outcomes and did not require additional intensive care assistance. Nevertheless, our findings raise concerns about viral evolution and the need for robust genomic surveillance. As a consensus, SARS-CoV-2 high transmissibility represents the key concern for the emergence of variants and selection of those who are adapted to escape the immune system and to spread more easily.

We detected both cases in two distinct places and the patients did not have any previous contact with each other, which led us to conclude that this viral strain was circulating in both places simultaneously. Importantly, mass gathering events places are crucial for the spread of respiratory viruses [16, 17], this added to the high mobility, and the spread of these viral strains is not surprising [18, 19]. Therefore, understanding and tracking these variants are necessary to improve the surveillance of the emergence of variants and/or mutations that might impact the diagnosis or clinical evolution of patients. This factor takes a special place today. Although the vaccination coverage is satisfactory in most places, many places, especially in Africa, still present low vaccination coverage rates (e.g. The Democratic Republic of Congo, which presents less than 6 doses per 100 habitants) [1]. The pandemic scenario is still concerning, and although less likely to appear new mutations/variants due to the considerable decrease in the number of cases, this emergence still might occur and become predominant by increasing the number of cases.

The failure to detect these mutations is concerning especially due to the mutation in binding sites of probes and primers should be considered for further testing, and kits should be constantly updated according to molecular epidemiological surveillance. In fact, by the beginning of the pandemic situation, this was a concern [20]. And more recently this is a matter that does not seem to be resolved. A previous study shows discordances in the testing available and approved by WHO [21]. We have observed the consequences related to the emergence of new variants, and their prompt detection and characterization are needed to avoid further issues. Therefore, persistent further studies are needed to determine the presence of strains like this one that might affect the efficacy of diagnostic methods.

Although the concerning facts related to the virus mutation, both patients presented good clinical evolution and outcome. Regardless of the fact one of them required hospitalization, the patients evolved without any further complications. Even though one of these patients did not have a travel history to places where SNV was more frequently found, our results highlight that the virus was probably circulating in Brazil for a considerable time before the first detection. And in fact, previous evidence indicates variants present a higher ability to escape the immune system [22], improve in binding properties of the virus on host cells [23] and become predominant over previous variants. This emphasizes the critical importance of ongoing surveillance and the regular updating of detection kits. Furthermore, it is imperative for countries to establish a genomic sequencing network to promptly generate alerts for epidemiological surveillance. These alerts can signal the emergence of variant strains with potential implications for public health. They enable governments to make informed decisions regarding whether to relax or maintain prevention and transmission control measures.

Finally, the SARS-CoV-2 pandemic condition was considerably controlled by the extensive vaccination program worldwide. But SARS-CoV-2 still presents a considerably high number of cases worldwide and many places still have low vaccination coverage.

## Data Availability

All relevant data are within the paper and supporting information files and details available upon request to João Renato Rebello Pinho.

## List of abbreviations

COVID-19: Coronavirus associated Disease 2019
ORF1ab: Conserved replicase domain
RT-PCR: Real time reverse transcription-polymerase chain reaction
SNV: Single Nucleotide Variant
ssRNA: Single-stranded RNA
UTR: 5’-untranslated region
